# Design and Manufacturing of Dielectric Resonators via 3D Printing of Composite Polymer/Ceramic Filaments

**DOI:** 10.3390/polym16182589

**Published:** 2024-09-13

**Authors:** Paris Sofokleous, Eva Paz, Francisco Javier Herraiz-Martínez

**Affiliations:** 1 Institute for Research in Technology (IIT), ICAI School of Engineering, Comillas Pontifical University, Santa Cruz de Marcenado 26, 28015 Madrid, Spain; eva.paz@iit.comillas.edu (E.P.); fjherraiz@icai.comillas.edu (F.J.H.-M.); 2 Mechanical Engineering Department, ICAI School of Engineering, Comillas Pontifical University, Alberto Aguilera 25, 28015 Madrid, Spain; 3 Electronics, Control and Communications Department, ICAI School of Engineering, Comillas Pontifical University, Alberto Aguilera 25, 28015 Madrid, Spain

**Keywords:** additive manufacturing, material extrusion, 3D printing, dielectric resonators, polymers, ceramics

## Abstract

Rapid technological advancements in recent years have opened the door to innovative solutions in the field of telecommunications and wireless systems; thus, new materials and manufacturing methods have been explored to satisfy this demand. This paper aims to explore the application of low-cost, commercially available 3D-printed ceramic/polymer composite filaments to design dielectric resonators (DRs) and check their suitability for use in high-frequency applications. Three-dimensional printing was used to fabricate the three-dimensional dielectric resonant prototypes. The filaments were characterized in terms of their thermal and mechanical properties and quality of printability. Additionally, the filaments’ dielectric properties were analyzed, and the prototypes were designed and simulated for a target frequency of ~2.45 GHz. Afterward, the DRs were successfully manufactured using the 3D printing technique, and no post-processing techniques were used in this study. A simple and efficient feeding method was used to finalize the devices, while the printed DRs’ reflection coefficient (S_11_) was measured. Results on prototype size, manufacture ease, printability, cost per volume, and bandwidth (BW) were used to evaluate the materials’ suitability for high-frequency applications. This research presents an easy and low-cost manufacturing process for DRs, opening a wide range of new applications and revolutionizing the manufacturing of 3D-printed high-frequency devices.

## 1. Introduction

The increasing development of new composite materials in additive manufacturing has revolutionized the scope of new applications of 3D printing [1]. In particular, emerging ceramic/polymer composites have opened the door for widespread, simple, and cost-effective 3D printing technologies such as fused deposition modeling (FDM), allowing for the printing of materials which, until recently, were deemed unfeasible with fusion-based technologies [2,3]. These composites are typically created by embedding ceramic particles within a polymeric matrix, enabling the flow of well-dispersed particles through the extruder during the melting process, thus facilitating the 3D printing of polymer/ceramic composites. In most applications, the polymeric matrix serves as a binder that is removed post-printing, leaving behind ceramic particles, which are then sintered to form the final ceramic material, being necessary for a post-treatment process of debinding and sintering [4,5]. However, these composite materials can also be leveraged in novel applications where such post-treatment is not required, such as in manufacturing scaffolds for bone regeneration [6,7]. This article proposes a new and promising application for these polymer/ceramic composite filaments in 3D FDM printing, unexplored until now and devoid of the need for debinding post-treatment: the design and manufacturing of dielectric resonators. Conventionally, these resonators are made using pure ceramic materials with high dielectric permittivity; however, conventional ceramic materials and manufacturing techniques present several disadvantages: (1) traditional ceramic forming methods like powder pressing and ceramic casting are limited in their ability to produce complex shapes cost-effectively; (2) the production cost of ceramic components is highly dependent on quantity, with small-scale production leading to high costs and long production times. The advent of the fused filament fabrication of ceramics (FFFC) offers a novel approach to overcoming these challenges [4,5].

The research of new materials and manufacturing methods for telecommunication and sensing applications has received great attention in recent years [8,9]. The demand for novel and high-performance dielectric resonators (DRs) is increasing exponentially, and the research community is forced to design new radiating and measurement systems to satisfy market demands. When R. D. Richtymer invented the term “dielectric resonator” and showed that non-metallic dielectric materials structures would act as resonators, it was a step forward in replacing metallic components and miniaturizing microwave circuits [10]. Their unique characteristics, like small size, light weight, ease of integration with planar transmission lines, and low loss, followed by high radiation efficiency due to the absence of conducting material, make DRs widely applicable for many applications [11]. In recent decades, they have been used in many microwave passive circuits, such as filters [12] and low-cost microwave signal generators, as oscillators [13]. Moreover, dielectric resonator antennae (DRAs) [14] have attracted attention in the literature, providing various antenna geometries which are practical for achieving a specific frequency response. Most recently, DR-based passive wireless sensors have also been reported [15]. Some applications of DR-based sensors are monitoring environmental conditions [16] and healthcare systems [17].

Nowadays, additive manufacturing (AM) is a widely popular technique for fabricating 3D electromagnetic (EM) structures in wireless systems, including antennae, sensors, graded-index lenses, etc. [18,19,20,21]. In particular, material extrusion is the most common 3D printing process and was introduced commercially in the early 1990s for various engineering applications [22]. Material extrusion is a rapid prototyping technology capable of printing high-resolution 3D parts. In terms of simplicity in printing, material extrusion is the most suitable technique over other 3D printing techniques, such as vat photopolymerization (VPP) and powder bed fusion (PBF), because it heats the filament to a semi-solid condition before placing it onto the print bed very easily. Furthermore, material extrusion 3D printers are the most economical and widely available [23]. For the purpose of this study, FDM, one of the prevailing methods of material extrusion, was employed. Leveraging the numerous advantages this technology offers, including adaptable unit sizes, user-friendly operation, and, notably, the low cost of machinery and feedstock materials [24], FDM was chosen for printing the prototypes. Direct ink writing (DIW) represents an alternative material extrusion method similar to FDM with many advantages, ideal for the fabrication of ceramics. DIW allows for the production of various sizes ranging from sub-micron to several millimeters to cater to diverse applications, including biomedical, packaging, and electronics, due to the flexibility of the process. However, carefully selecting process parameters and ink additives requires thorough optimization and extensive trial and error, which adds complexity and reduces feasibility [25]. Additionally, this approach tends to be more costly than FDM.

Conventional DRs, like antennae, suffer limitations in radiation efficiency and impedance bandwidth (BW) because of their size, the material’s dielectric properties, and less design freedom [14,26]. However, as a low-cost printing technique, AM could provide new dielectric structures. Due to its capability of fabricating complex shapes and printing with fast and precision prototyping, and the use of polymer/ceramic composites could bring a breakthrough in this field. The necessity of low-cost products, fast manufacturing processes, and easy-to-use techniques is more demanding, and the material extrusion 3D printing technique can, undoubtedly, offer all these benefits [27].

Recently, some ceramic materials have been utilized in 3D printing to fabricate dielectric structures [28,29,30,31]; however, the materials examined in this study, such as zirconia (ZrO_2_), hydroxyapatite (HA), and titanium oxide (TiO) materials, have not previously undergone testing for DR applications. Taking advantage of their excellent properties, ceramics are used in a wide range of engineering applications, characterized by high permittivity (ε) and thus high dielectric constant and low loss tangent (tan δ). Due to their high ε, they are excellent materials for designing DRs since very small devices with a high quality factor (Q factor, the inverse of the −3 dB BW) can be realized. However, they have some limitations, such as being brittle and having poor tensile strength. At the same time, pure polymers are not usually used for printing dielectric structures because they have very low ε (leading to huge dimensions) and high losses (high tan δ). Therefore, ceramics mixed with thermoplastic polymers such as polylactic acid (PLA) and acrylonitrile butadiene styrene (ABS) seem an attractive solution for manufacturing these dielectric structures using AM techniques [32].

First, the filaments were characterized for their thermal behavior, glass transition, and melting temperature to ensure proper application in 3D printing. Pure polymer materials have been used for comparative purposes. Moreover, their mechanical properties determine the tensile properties of each material, using dog-bone-shaped specimens for these tests. In addition, the quality of the printability was evaluated by quantifying the porosity in the printed materials resulting from printing imperfections, and the presence of defects was also evaluated with an optical microscope. Furthermore, the dielectric structures have been designed in a low GHz band (2.45 GHz). The targeted frequency, 2.45 GHz, is the most-designated ISM (industrial, scientific, medical) frequency band for wireless networking. The dimensions of each DR, depending on the material’s dielectric constant, determined the structure’s size; thus, they were computed and simulated in a full-wave electromagnetic (EM) simulator. Therefore, all the prototypes were printed, and the power reflection coefficient of the printed prototypes was measured. Moreover, this study did not use post-processing techniques to simplify and reduce fabrication costs. In the end, all the 3D printing materials were compared in terms of their dimensions, cost, printability, and BW to evaluate their potential for high-frequency applications. In particular, as commented before, DR-based sensors are a novel technology with several applications. For this reason, the final comparison and application will be focused on the possibility of making 3D-printed DR sensors.

This study aimed to harness the potential of the application of commercially available 3D printing ceramic/polymer composite filaments reinforced with high doses of ceramic fillers as a promising alternative to the current manufacturing methods and materials used for high-frequency applications. It should be emphasized that the particular application of fabricating DRs has not yet been explored in the existing literature. A final comparison will be presented to study the suitability of the considered materials for different high-frequency applications.

## 2. Materials and Methods

### 2.1. Materials

This study used different polymeric filaments, pure and reinforced with ceramic fillers. As pure polymers, polylactic acid (PLA) (PRUSA, Prague, Czech Republic), acrylonitrile–butadiene–styrene (ABS) (Filament2print, Pontevedra, Spain), and polyolefin (PO) (Nanoe, Ballainvilliers, France) were tested. The polymer composites selected for this study have been categorized according to the ceramic filler and the polymeric matrix, as shown in Table 1. Cooling was enabled, but retraction was not utilized with the 3D printer. Furthermore, the printed components were produced on a glass bed.

### 2.2. Three-Dimensional Printing Process

A commercial 3D printer (PRUSA i3 MK3S, Prague, Czech Republic) was used to produce all the samples (bricks, dog-bone specimens) and prototypes (DRs). First, slicer software (PrusaSlicer version 2.8.0) was used to adjust the printing parameters for each material, such as the printing/bed temperatures, print speed, layer height (0.2 mm), nozzle diameter, etc. All specimens and prototypes were optimized to print with 100% of infill density. The program enables the conversion of STL files into the G-Code programming language for the 3D printer. Before printing, it was necessary to find the optimal parameters for each material, and the filaments must be pre-dried. The polyolefin-based material filaments had a higher filler loading and could not be printed with a 0.6 mm nozzle diameter, forming some agglomerates inside the extruder, preventing it from printing; therefore, they were optimized for use with a 0.8 mm nozzle. Table 2 shows the optimized printing parameters used in each case.

### 2.3. Differential Scanning Calorimetry (DSC)

A dynamic differential scanning calorimetry (DSC) analysis was performed on a DSC equipment DSC822 (Metter, Madrid, Spain) instrument under a nitrogen flux of 80 mL/min, used as a purge gas, applying a heating scan between 20 and 300 °C at 20 °C/min. DSC tests were conducted using an aluminum crucible with a capacity of 40 µL; the weight of samples tested was between 5 and 10 mg. DSC evaluated all the filaments to determine their suitability for the 3D printing procedure based on their glass transition temperature (T_g_) and melting point (T_m_) to facilitate the optimization of the printing temperature. StarSystem software 2004 (Mettler Toledo, Madrid, Spain) was used to evaluate the results. In each experiment, three different samples of each material have been tested, facilitating the computation of the average values for both T_g_ and T_m_ for each filament. The T_g_ was calculated as the midpoint.

### 2.4. Tensile Tests

Tensile tests were performed using a Universal Testing Machine IBTH 500 (Ibertest, Madrid, Spain) following ISO 527-2:2012 standard indications [33]. Three dog-bone shape specimens of each material with dimensions of 75 × 10 × 2 mm (height × width × thickness) were tested to investigate their tensile properties. The samples were manufactured using the Prusa 3D printer. The crosshead speed employed during the tests was 3 mm/s. The mechanical properties of the materials—Young’s modulus, tensile strength, and maximum deformation at break—were determined by analyzing the obtained stress–strain curve. The results were obtained from the average values of the three specimens of each material.

### 2.5. Density Tests

The density tests were performed according to Archimedes’ principle using a balance. Three 3D-printed bricks of each material with dimensions of 25 × 30 × 5 mm, 38 × 30 × 5 mm, and 55 × 30 × 5 mm (height × width × thickness) were manufactured for density analysis. Furthermore, the density of each filament before printing was also determined for comparative purposes. The testing was carried out using distilled water as an immersion liquid for all the bricks at room temperature, except for PO (due to its lower density than water), where ethanol was used to carry out the test. According to Equation (1), the total density was measured by the mass of samples in the air (m_a_), which was initially determined before and after immersing into the liquid (m_b_), the density of the water (at room temperature) ($\rho_{w}$), and the air density ($\rho_{a}$). The tests were repeated two times for the bricks and three times for the filaments, and the average value was recorded. The porosity (%) of the bricks was calculated with Equation (2), using as a reference the filament porosity.

(1) $$\rho =\frac{m_{a}}{m_{a}-m_{b}}\left(\rho_{w}-\rho_{a}\right)+\rho_{a}$$

(2) $$\mathrm{Porosity}\;\left(\%\right)=\left(1-\frac{\rho_{\mathrm{brick}}}{\rho_{\mathrm{filament}}}\right)*100$$

### 2.6. Microscopy Analysis

To investigate the influence of the printing process on the 3D-printed parts and evaluate the printing quality, the printed samples were examined by opto-digital microscopy with an Olympus DSX1000 (Olympus, Tokyo, Japan).

### 2.7. Dielectric Characterization

For the dielectric characterization, a split-post dielectric resonator (SPDR) (QWED, Warsaw, Poland [34]) was used for measuring the relative permittivity (ε_r_) and the dielectric tan δ of laminar dielectric 3D-printed materials. SPDR functions as a resonant cavity equipped with a slot designed for the insertion of samples. In principle, 3D-printed bricks for each studied material with dimensions of 60 × 100 × 2 mm (height × width × thickness) were manufactured to be tested on the SPDR. A microwave signal generator was essential for assessing the transmission (|S_21_|) characteristics between the two ports of the SPDR and extracting the resonant frequency and Q-factor. To fulfill this requirement, a vector network analyzer (VNA) (Anritsu MS46122B, Atsugi, Japan [35]) operated as a signal generator capable of measuring transmission characteristics during the SPDR measurements of the 3D-printed brick samples. VNA is an instrument designed to gauge the frequency response of either a single component or a network consisting of multiple components. It records both the amplitude and phase of the high-frequency signal at various frequency points [36]. Nowadays, VNAs are used in a wide range of radio frequency and high-frequency applications. More specifically, it compares the incident signal that leaves the analyzer with either the signal that is transmitted through the test device (|S_12_|, |S_21_|) or the signal that is reflected from its input (|S_11_|, |S_22_|). Moreover, before measuring, a three-term calibration had to be performed [37]. The VNA was calibrated between 2 and 3.5 GHz. To perform the dielectric characterization, the SPDR method requires two consecutive measurements: one for the empty resonator (air) and one for the resonator with the brick sample. The resonant frequency and Q factor of the empty resonator were measured and recorded [34,38]. This process was replicated for every brick sample. The resonant frequency and Q factor of each transmission measurement (|S_21_|) were obtained. Subsequently, specialized software (QWED, Q-meter 2019, Warsaw, Poland [34]) was employed to calculate each printed sample’s ε_r_ and dielectric tan δ values based on the transmission measurements and the height of each brick.

### 2.8. Design and Manufacturing of DRs

The DRs were designed and simulated in CST Studio Suite (Dassault Systems, Paris, France [39]), an electromagnetic simulation program for designing, analyzing, and optimizing electromagnetic (EM) components and systems. The DR’s prototypes were designed for a target frequency of ~2.45 GHz, using the ε_r_ and loss tan δ obtained from the previous section. The parameters of each design were optimized depending on their dimensions and ε_r_ values. The frequency range used for the simulation was 2–3.5 GHz. Regarding the shape of the DR, a cylinder was chosen because it is the most used geometry for DRs and has the ease of fabrication [14]. The fundamental mode of each resonator was selected, which is the one with the lowest resonant frequency. It was necessary to design an adequate feeding strategy to excite the resonators. Dealing with the feeding technique, the probe-fed configuration, with the probe penetrating the DR, was selected for this kind of application due to its ability to achieve high coupling to the DR, and the resonance frequency can be regulated [14].

**Figure 1:**
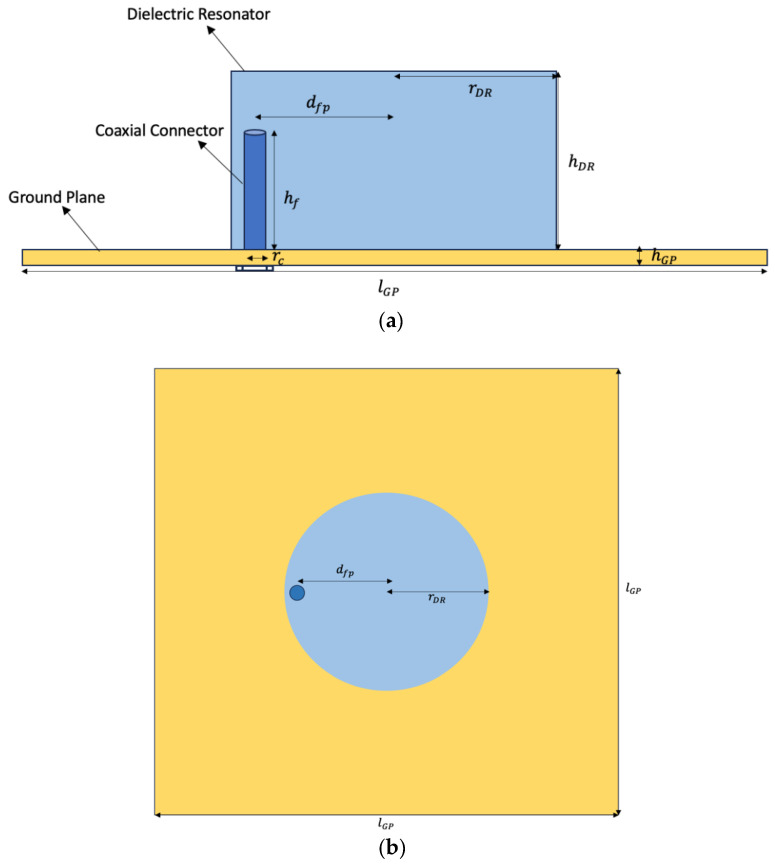
DR prototypes: (**a**) side view; (**b**) top view. DR’s radius (r_DR_); DR’s height (h_DR_); ground plane’s length (l_GP_); ground plane’s height (h_GP_); feeding point distance (d_fp_); feed height (h_f_); coaxial radius (r_c_).

Moreover, this feeding technique is easy to implement and cheap, contrary to other feeding techniques. Regarding the manufacturing of the feeding implementation, copper ground planes (GPs) were drilled, forming a hole at a distance from the plane’s center, depending on each design’s optimal feeding point parameter. The hole was 5 mm in diameter to insert the inner pin of the coaxial connector (RS Components, female type, Corby, UK) inside and fix the outer part of it on the GP. Afterward, glue (Loctite 406, Henkel Iberica, Madrid, Spain) was used to set the connector in the center of the hole, adding silver ink (RS Components, Corby, UK) above the outer part of the connector. Afterward, to check the conductivity between the GP and the connector. The side and top view of the prototypes, as well as their design parameters, are shown in Figure 1. After manufacturing the final prototypes and fixing the GPs with the coaxial connectors, the DRs were placed on top of the connectors, and the resonant frequency and the reflection coefficient were measured.

### 2.9. Frequency Response and Bandwidth of the DRs

The DRs placed on the ground planes were connected to port 1 of the VNA. This study allowed us to measure the frequency response of the printed DR prototypes. As one-port devices, the measured frequency response is the reflection coefficient |S_11_|. Therefore, the minimum of the reflection coefficient (|S_11_|), the resonant frequency (f_0_), and the −10 dB BW were calculated for all the DRs from the measurements. The −10 dB BW was obtained from Equation (3), where f_max_ and f_min_, the maximum and minimum frequency at −10 dB, respectively, and f_0_, the resonant frequency of each DR prototype, were obtained.

(3) $$\mathrm{BW}\;\left(-10\mathrm{dB}\right)=\frac{f_{\max}-f_{\min}}{f_{0}}$$

## 3. Results and Discussion

### 3.1. Thermal Analysis

The thermal analysis of the filaments is essential to understand the polymer composite behavior during the heating process thoroughly and, in this case, to determine if the presence of ceramic fillers will affect the printing parameters.

**Figure 2:**
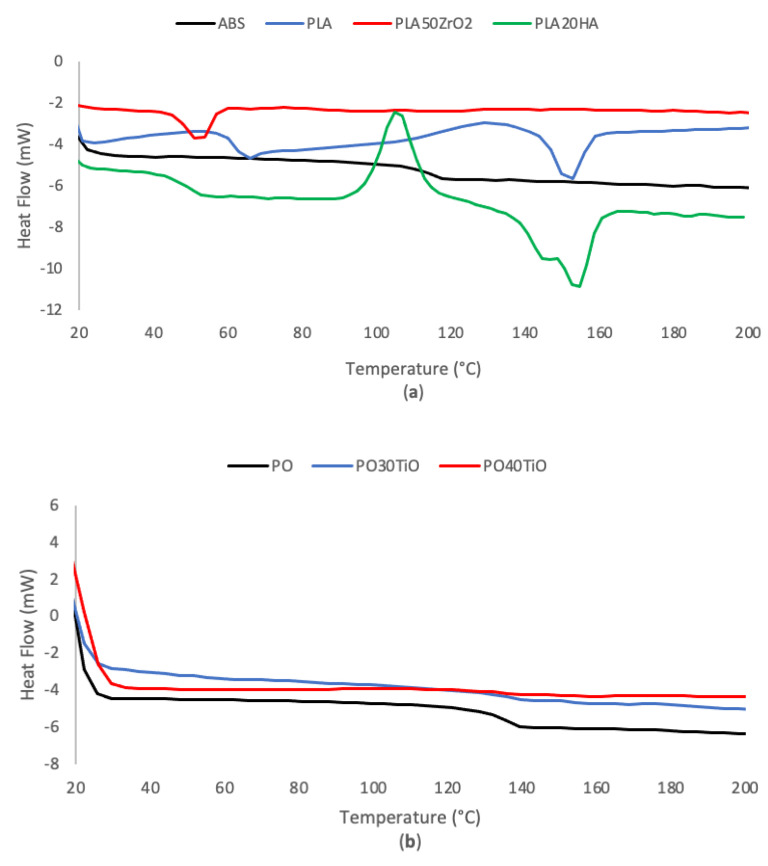
DSC curves for (**a**) ABS, PLA, PLA50ZrO_2_, and PLA20HA; and (**b**) PO, PO30TiO, and PO40TiO.

Table 3 summarizes the results of every material used for the study regarding their glass transition temperature (T_g_) and melting point (T_m_) after the second heating scan. In all cases, the results did not exhibit significant differences between the two scans, although the T_g_ and T_m_ of the samples in the second scan slightly decreased compared to the first scan. Figure 2 illustrates the experimental DSC curves for each tested material. Overall, the T_g_ of ABS was higher than PLA (111.4 °C and 58.8 °C, respectively), while the T_g_ of PO showed the highest value of all pure polymers (135.0 °C). In the case of the T_g_ of the PLA-based ceramic filaments (HA and zirconia), PLA20HA slightly decreased (46.6 °C) in comparison to PLA (58.8 °C), while the T_g_ of PLA50ZrO_2_ (58.5 °C) has a similar value to the one of PLA. However, neat PO and PO-based materials (PO30TiO and PO40TiO) have the highest T_g_ values. Contrary to what was observed in the other polymers, in the case of PO40TiO, the presence of TiO ceramic particles seems to increase the T_g_ of the polymer slightly. Regarding the T_m_, PLA, and PLA20HA pointed out the same melting temperature (around 150 °C); but on the other hand, the rest of the materials did not exhibit any melting peak. The reason is the very high amount of amorphous material; therefore, they do not undergo melting as they do not have a crystalline structure.

The glass transition temperature (T_g_) of polymer composites containing ceramic fillers is influenced by factors like particle size, the interaction with the polymeric matrix, and the hydrophilicity of the filler. For instance, previous studies have demonstrated that the presence of HA can induce partial hydrolysis of the PLA chains due to its highly hydrophilic nature [40]. This hydrolysis promotes the plasticization of PLA oligomers, resulting in a decrease in T_g_. These effects are complex, but from a practical perspective, it is crucial to understand how these temperature variations can impact the printing process, as it may require more or less heat to achieve the appropriate viscosity of the polymer during printing. Finally, these results helped obtain the optimal printing parameters of Table 2, where, in the end, the optimal printing temperatures were lower than the ones used during the printing process.

### 3.2. Mechanical Properties

**Figure 3:**
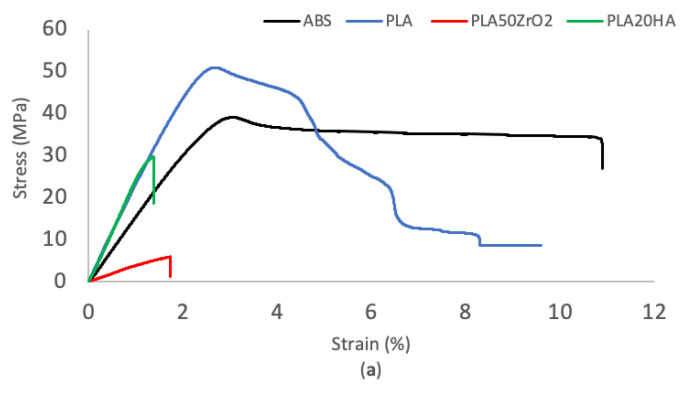

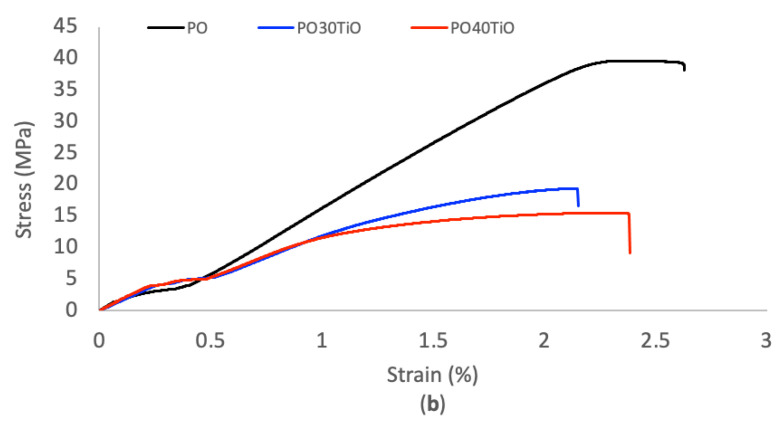
Stress–Strain curves for (**a**) ABS, PLA, PLA50ZrO_2_, and PLA20HA; and (**b**) PO, PO30TiO, and PO40TiO.

The graphical representation of the stress–strain curves is essential to evaluate a material’s mechanical response. Figure 3 shows one representative experimentally measured curve for each tested material. In Figure 3a, ABS demonstrates a lower tensile strength, deformation, and Young’s modulus than pure PLA, as indicated in Table 4. Moreover, the samples produced from PLA with ceramic fillers (HA and zirconia) also exhibits a lower tensile strength (29.4 and 4.2 MPa, respectively) and deformation (1.3 and 2.7%, respectively) than samples fabricated from neat PLA (48.4 MPa and 9.9%), as shown in Figure 3a and Table 4. This was especially noticeable in PLA50ZrO_2_ specimens since they also have a much higher volume of ceramics (50%) than PLA20HA (up to 20%). Notably, the Young’s modulus values of neat PLA and PLA20HA are very similar. Figure 3b shows the stress–strain curve of PO-based materials reinforced with TiO. In Table 4, it can easily be observed that the results are very similar, but in the case of PO40TiO, the values are slightly lower compared to PO30TiO. The same applies in this case since PO40TiO contains a higher volume of ceramic filler than PO30TiO.

Adding ceramic fillers significantly affects the mechanical properties of tensile specimens compared to pure polymers. The higher the volume percentage of incorporated fillers in the matrix, the more the mechanical properties of the material decrease. The mechanical properties of the material, including ultimate tensile strength, maximum deformation, and Young’s modulus, can be influenced by adjusting parameters such as particle size, particle loading percentage, and interfacial adhesion/area. This impact occurs through the modulation of polymer chain mobility by increasing the ceramic content in the matrix [41,42]. It is crucially important to take this effect on mechanical properties into account when evaluating the final feasibility of a design made with this type of material since, in some instances, mechanical stability could be compromised.

### 3.3. Printability

Density is one of a material’s most notable and easily measured physical properties. The average density results obtained from filaments and printed bricks for each material are shown in Table 5 as the density loss percentages after the printing process (filaments vs. bricks). The measurement of the density can provide an idea of the quality of the printed pieces since, being printed with 100% infill, a greater porosity can, in part, indicate the presence of printing defects, poor deposition, and adhesion of the layers or the presence of gaps, creating zones of free volume.

**Figure 4:**
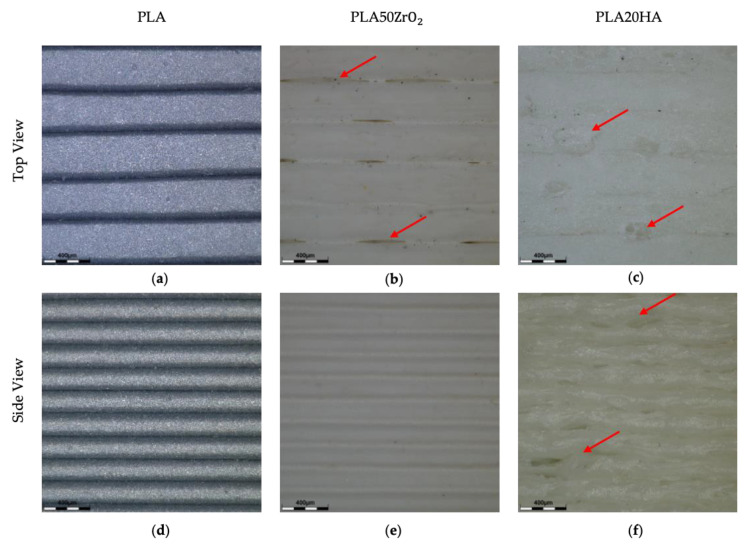
Top and side view images of 3D-printed bricks: PLA (**a**,**d**), PLA50ZrO_2_ (**b**,**e**), and PLA20HA (**c**,**f**), respectively. Arrows indicate the most notable defects.

As shown in Table 5, pure polymers (ABS, PLA, PO) and the two PO-based materials (PO30TiO and PO40TiO) revealed comparable porosity after printing. The most noteworthy detail is that PO30TiO indicated only 0.07% of porosity. Additionally, PLA50ZrO_2_ indicated a high porosity (4.58%), while PLA20HA showed a significantly higher porosity (10.77%). As shown in the optical microscope concerning the top-view images in Figure 4, PLA (a) displayed high print quality with uniform layers and well-bonded lines. In contrast, PLA50ZrO_2_ (b) exhibited some defects between printed lines, while PLA20HA (c) had voids and bubbles on the top layer. Notably, the print quality of other materials was generally regular and flat, with low-density losses likely due to thermomechanical loading during manufacturing [43]. In the side-view images of the 3D-printed bricks, PLA (d) and PLA50ZrO_2_ (e) demonstrated good printability with consistent layer deposition and minimal defects. However, PLA20HA (f) exhibited significant voids, air gaps, and defects, leading to reduced density and affecting other material properties, such as ε_r_ which is critical for resonator design, as discussed in subsequent sections. The observed differences in porosity between PLA50ZrO_2_ and PLA20HA can be attributed to their print quality; while PLA50ZrO_2_ had minor defects on the top layer, its side view showed well-printed, regular layers. In contrast, PLA20HA displayed irregular layer deposition and gaps, likely due to its high viscosity during the 3D printing process.

The manufacturing process plays a vital role in the 3D printing structures since parameters such as printing/bed temperature, nozzle size, layer thickness, printing speed, etc., can greatly affect the final printed parts directly. However, the material properties, filler composition in the polymer matrix, and printability contribute to the formation of air gaps and defects, leading to high porosity (density loss). Materials with ceramic fillers are more brittle and challenging to print. Warping issues arise in some materials (ABS, PO, PO30TiO, and PO40TiO) due to shrinkage. Additionally, moisture can affect printability, often causing bubbles and holes in the extruded filament during the printing process as well.

### 3.4. Electromagnetic Measurements

#### 3.4.1. Dielectric Results

Table 6 presents the ε_r_ and dielectric loss tangent values for each printed brick measured using SPDR and Q-meter tests. According to the results, PLA50ZrO_2_ has the highest ε_r_ among all the materials, largely due to its higher ceramic filler content, while neat PLA and ABS materials exhibit the lowest (~3), consistent with references [44,45,46]. On the other hand, PLA and PLA20HA show the highest dielectric losses. In contrast, the PO-based materials have significantly lower losses, making them particularly promising for certain EM applications.

It is worth mentioning that in the case of pure PO material, the ε_r_ was <2. Thus, it was not a suitable material for fabricating a prototype from an EM perspective because it is very close to the ε_r_ of the vacuum and would lead to huge DRs dimensions. For microwave sensing applications, high ε_r_ is essential and directly related to small-size devices, while, on the contrary, low ε_r_ is related to big dimension devices. Dielectric losses also affect the permittivity and therefore the final device’s dimension. From an EM point of view, PLA50ZrO_2_ and PO40TiO are the most notable materials for this kind of application, as Table 6 shows.

#### 3.4.2. DRs Design and Prototypes

Table 7 indicates the parameters that were processed and optimized for the DR designs to achieve the target frequency of approximately 2.45 GHz and the minimum reflection coefficient (|S_11_|) of the resonators. The copper ground planes used in the designs had uniform dimensions of 200 mm in length and 1 mm in height across all prototypes. The prototypes were designed for a target frequency of around 2.45 GHz, with their dimensions largely determined by the material’s dielectric constant ε_r_. Additionally, Table 8 presents the experimental results obtained from the VNA measurements, including the resonant frequency, the minimum of the reflection coefficient (|S_11_|), and the −10 dB BW.

**Figure 5:**
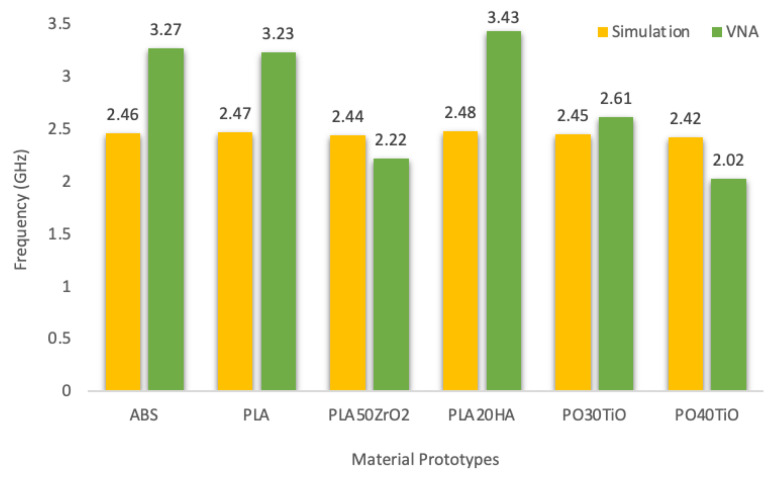
Theoretical (simulation)–Experimental (VNA) resonant frequency of the prototypes.

The results indicate a distinct shift in frequency between the simulation and VNA (Figure 5). Materials without ceramic content, such as ABS and PLA, as well as those with relatively low content (PLA20HA), indicated a more significant frequency shift compared to PLA reinforced with zirconia and PO-based materials (PO30TiO and PO40TiO), which exhibited only a minor shift. This is primarily attributed to the effective ε_r_ of the prototypes, a weighted average between the air’s ε_r_ and the material’s ε_r_. Prototypes with lower effective ε_r_ tend to be bulkier and more porous, making the air content significant. Higher porosity leads to lower effective ε_r_, resulting in higher frequencies and broader BWs. This is something to be considered because the ε_r_ results were taken from the 3D-printed bricks and do not directly represent the ε_r_ of the prototypes. However, despite PLA20HA having a similar ε_r_ to PO30TiO (3.66 and 3.64, respectively) and relatively higher ε_r_ than the pure polymers, it still exhibited a significant frequency shift. This may be due to PLA20HA containing a lower ceramic filler content (HA up to 20%) compared to the other materials (TiO at 30% and 40%, zirconia at 50%), as well as its very high porosity (10.77%), which results in higher measured resonant frequencies.

Conversely, materials with a high amount of ceramic content (PLA50ZrO_2_, PO40TiO), and thus higher ε_r_, produce small-size prototypes with a narrow BW and minimal frequency shifts. It is worth noting that PLA50ZrO_2_ and PO40TiO demonstrated a slightly lower resonant frequency than the other prototypes in the VNA measurements. This is likely due to their higher effective ε_r_. The significant ceramic content in these prototypes results in an effective ε_r_ that is much higher than that of the 3D-printed bricks’ ε_r_, leading to a lower resonant frequency in the VNA compared to the simulated values. This indicates that these DRs have a significantly smaller volume for the same frequency band. As a result, there are fewer errors and less trapped air within the structure during printing, leading to improved printability. Additionally, frequency shifts are smaller in DRs when using materials with higher permittivity. Since the resonant frequency is inversely proportional to $1/\sqrt{\varepsilon_{r}}$, a small variation in ε_r_ when ε_r_ is high results in minimal changes to the frequency.

PO30TiO indicated a minor shift as well but, in this case, with a broader BW (36%) than PLA50ZrO_2_ (17%) and PO40TiO (16%). The reason is that in VNA measurements, the PO30TiO prototype (as well as the PLA and PLA20HA prototypes) exhibited two overlapping resonances instead of a single one, resulting in a broader BW. Notably, while the PO40TiO prototype is not much smaller than the PO30TiO, the difference in their ε_r_ values is significant. Even though PLA20HA is more compact, it has a lower ε_r_ compared to PO40TiO. This discrepancy is also attributed to feeding parameters such as the d_fp_ and the h_f_ used during the design of the prototypes, which influence the final dimensions of the design. It is also worth mentioning that the minimum of the reflection coefficient of these resonators, obtained from VNA measurements, was still low (indicating good matching), making them very interesting for certain high-frequency applications.

#### 3.4.3. Comparison and Potential Applications

**Figure 6:**
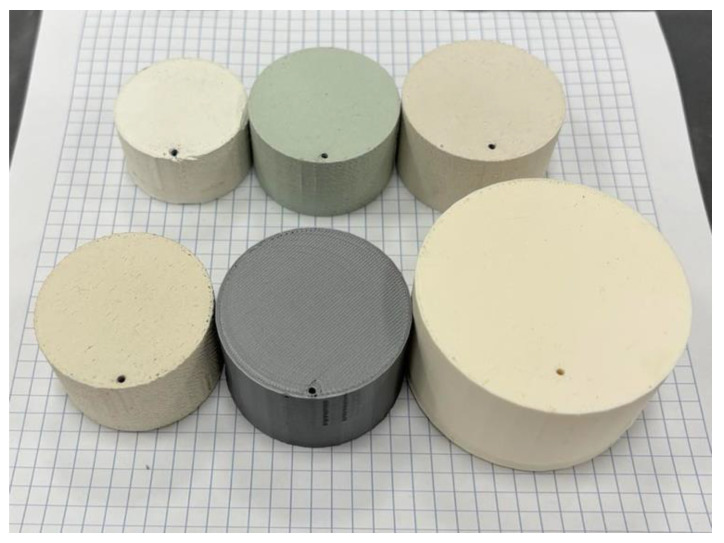
The 3D-printed prototypes. From top left to bottom right: PLA50ZrO_2_; PLA20HA; PO30TiO; PO40TiO; PLA; and ABS.

In this section, Table 9 is referenced to compare the materials used in the experiments, the dimensions of the resonator prototypes, manufacturing ease, and printability. The cost of material filaments per prototype volume measured in millimeters (mm) and wavelength (λ) and the −10 dB BW are also discussed to identify the most suitable materials for high-frequency applications. Figure 6 clearly shows that the PLA50ZrO_2_ prototype has the smallest volume, while the ABS prototype has the largest. According to Table 6, and in conjunction with the results in Table 9, materials with higher ε_r_ result in smaller prototypes, whereas those with lower ε_r_ yield larger resonant devices. Regarding the printability of each material, using 3D printing technology, the pure polymer filaments (PLA, ABS) were the easiest to print without significant issues. Maintaining lower, consistent temperatures is crucial for zirconia and HA in the PLA matrix to prevent nozzle clogging from agglomerates. PO-based materials require higher temperatures and slower printing, along with pre-heating to avoid warping. While pure polymers remain the easiest to print, 3D printing with ceramics, though more complex, is feasible and can produce high-quality prints with the right parameter adjustments. However, there may be limitations when working with complex geometric designs. In terms of cost, common 3D printing filaments like pure ABS and PLA are very affordable, priced at 2.22 EUR and 1.39 EUR per prototype, respectively. Filaments with ceramic fillers vary in price: PO30TiO is relatively inexpensive at 19.08 EUR/prototype, while PO40TiO costs around 30 EUR/prototype. Despite containing 50% ceramic, PLA with zirconia remains cost-effective at 58.75 EUR/prototype. However, PLA20HA is quite expensive at 212.86 EUR/prototype, which renders PLA20HA a high-priced filament compared to the other material filaments. Overall, it is essential to use low-cost, easily printable materials and produce small, manufacturable devices for mass applications.

**Figure 7:**
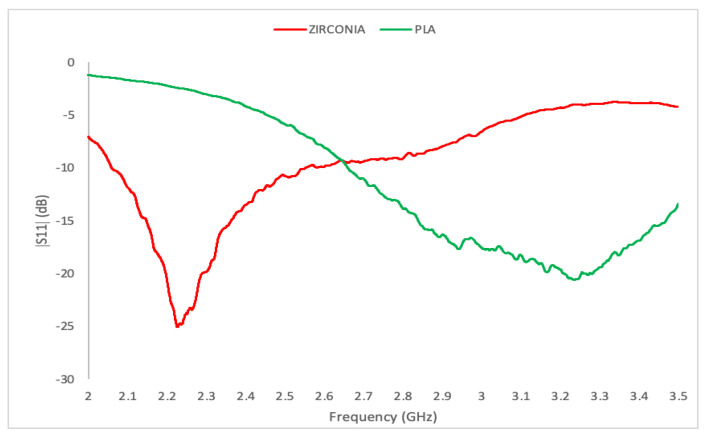
Frequency response—Minimum of reflection coefficient (|S_11_|) between PLA50ZrO_2_ and PLA prototypes.

Electromagnetic results categorize materials by ε_r_ in different applications. For instance, low ε_r_ materials are ideal for DRA applications [47] due to their broader BW. High ε_r_ materials are excellent for sensing applications, especially as sensors [15], offering narrow BW, high sensitivity, small size, and lower cost. Figure 7 shows the frequency response and the minimum of reflection coefficient (|S_11_|) between PLA50ZrO_2_ and PLA prototypes. It is obvious that neat PLA indicated a broader BW and resulted in higher frequency than PLA50ZrO_2_. The results have shown that since all the prototypes were designed to operate at approximately the same frequency (~2.45 GHz), the BW and the frequency shift are higher in polymers compared to ceramics. Ultimately, all the materials demonstrated feasibility for this innovative application, where 3D printing has yet to establish a strong presence, offering highly promising possibilities.

**Figure 8:**
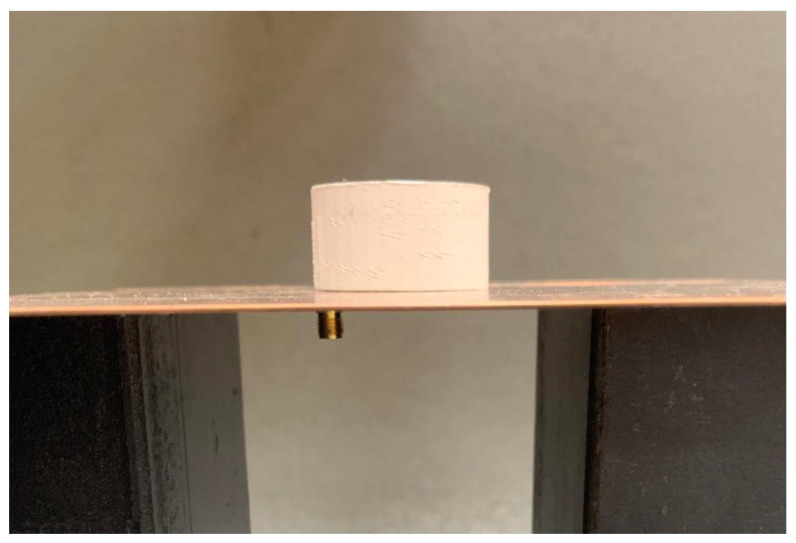
PLA50ZrO_2_ prototype on the GP, fed by the coaxial connector.

**Figure 9:**
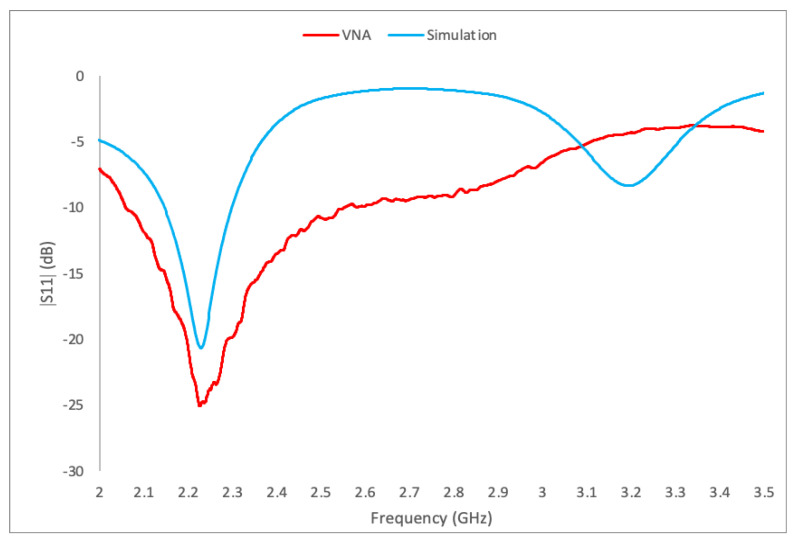
Simulated and measured frequency response and reflection coefficient (|S_11_|) of PLA50ZrO_2_ prototype.

Based on simulations, experimental results (VNA), and data from Table 9, PLA50ZrO_2_ emerges as the most suitable material for sensing applications. Undoubtedly, it has the higher ε_r_ among the materials tested and produces the smallest DR prototype. Hence, it offers a narrow BW with a low reflection coefficient, enabling more precise measurements with high sensitivity, making PLA50ZrO_2_ an ideal material for a sensing application. Additionally, it provides a good trade-off between printability and cost, though it has poor mechanical properties. Figure 8 shows the PLA50ZrO_2_ prototype mounted on the ground plane, and Figure 9 illustrates the simulation (CST) and experimental (VNA) curves of the frequency response and minimum of |S_11_| of that prototype. The resonant frequency was initially simulated at 2.44 GHz with the minimum |S_11_| at −23 dB, but VNA results indicated a resonant frequency of 2.22 GHz and minimum |S_11_| at −25 dB.

The prototype was re-simulated to match the experimental frequency, as shown in Figure 9. The updated simulation accurately matched the experimental frequency of 2.22 GHz, with a minimum |S_11_| at −21 dB. The new simulated ε_r_ was 8.5, slightly higher than the measured ε_r_ of 8.16 (bricks). This increase is expected because the initial simulated frequency was 2.44 GHz, and to align with the lower experimental frequency of 2.22 GHz, the effective ε_r_ needed to be higher in this case. In addition to porosity and ceramic content affecting permittivity and frequency shift, other factors contribute to the discrepancies between the initial simulations and experimental results. For instance, tolerances in 3D printing and simulation can lead to differences in prototype dimensions. Additionally, the impact of air gaps between the GP, connector, and DR is also noticeable and affects the prototype’s effective ε_r_.

Recently, DRs have been utilized in sensing applications [15,48,49]. Most of these studies have employed conventional manufacturing techniques. The proposed PLA50ZrO_2_ prototype exhibits similar characteristics to those reported in relevant studies and benefits significantly from 3D printing. The proposed prototype performed well, with low reflection coefficients and a narrow BW. Furthermore, the DR feeding was both simple and effective. The printability was satisfactory, and the cost per unit was affordable. These attributes make it an excellent candidate for future DR-based sensors. Nevertheless, it is worth mentioning that HA and zirconia have been outstanding materials for biological applications [50]. Due to their desirable dielectric and printing properties, HA’s excellent bioactivity and biocompatibility, and zirconia’s bioinert characteristics, both materials are strong candidates for future biological sensing devices.

## 4. Conclusions

The present research study aimed to design and develop DR prototypes using 3D printing commercial ceramic/polymer composite filaments, assessing their suitability for high-frequency applications. The material filaments were successfully characterized in terms of their thermal behavior, the mechanical stability determining their tensile properties, and the quality of their printability. Thermal results estimated the feasibility of the filaments to be used in 3D printing by optimizing the printing temperature. Mechanical results have shown that the incorporation of ceramic fillers significantly affects the materials’ mechanical properties, which is crucial for prototype manufacturing and their final application. Additionally, the printability of the materials, and therefore the porosity, massively affects the ε_r_ and mechanical properties of the materials and, consequently, the frequency response of the final prototype. Pure polymer filaments like ABS and PLA were the easiest to work with, showing no significant issues during the printing process. However, although printing with ceramic-filled materials such as zirconia and hydroxyapatite in the PLA matrix is more challenging, keeping consistent temperatures is essential to prevent nozzle clogging caused by agglomerates, and they can offer high-quality prints by adjusting their parameters. Moreover, ceramic materials have shown good printability through 3D printing, which opens a wide range of new applications utilizing these kinds of materials and revolutionizing additive manufacturing.

From a cost perspective, pure ABS and PLA filaments were the most affordable, while filaments with ceramic fillers were relatively more expensive. PLA50ZrO_2_ was found to be cost-effective at EUR 58.75 per prototype, making it viable for mass applications, while on the other hand, PLA20HA was the most expensive at EUR 212.86 per prototype, making it less viable for mass production.

Following the dielectric characterization, the prototypes showed very low reflection coefficients, while FDM 3D printing facilitated the cost-effective production of DR prototypes without requiring post-processing. Particularly, the PLA50ZrO_2_ prototype achieved a simulated resonant frequency of 2.44 GHz and a measured frequency of 2.22 GHz, with a minimum reflection coefficient of −25 dB, indicating good matching and minimum reflection losses. The results highlighted that ceramic-filled materials, despite their printing challenges, can produce high-quality DRs with excellent electromagnetic performance. The findings indicated that materials with higher ε_r_ and ceramic content in the polymer matrix yield small sizes and narrow BWs, ideal for sensing applications. Conversely, low ε_r_ materials like pure polymers produce larger sizes and broad BWs, suitable for broadband applications like DRAs.

In summary, the research demonstrated that polymer/ceramic composites are highly suitable for high-frequency sensing applications due to their high dielectric constant, compact size, and narrow BW. This study also underscored the feasibility of using 3D printing to manufacture complex DR structures, opening new possibilities in both high-frequency and biological sensing applications. Among the materials tested, PLA50ZrO_2_ emerged as a leading candidate for sensing applications, balancing cost-effectiveness with performance.

## Figures and Tables

**Table 1:** Polymer composites were used for the study based on their polymeric matrix, reinforced fillers, and composition.

Materials	Polymeric Matrix	Ceramic Filler	Filler Content	Supplier
PLA50ZrO_2_	PLA	Zirconia (ZrO_2_)	50%	Zetamix, Nanoe, Ballainvilliers, France
PLA20HA	PLA	Hydroxyapatite (HA)	Up to 20%	COLFEED4Print, Madrid, Spain
PO30TiO	Polyolefin (PO)	Titanium oxide (TiO)	30%	Zetamix, Nanoe, Ballainvilliers, France
PO40TiO	Polyolefin (PO)	Titanium oxide (TiO)	40%	Zetamix, Nanoe, Ballainvilliers, France

**Table 2:** Printing parameters of the materials.

Materials/Properties	Printing Temperature (°C)	Bed Temperature (°C)	Print Speed (mm/s)	Nozzle Diameter (mm)
ABS	255	100	80	0.6
PLA	215	60	80	0.6
PLA50ZrO_2_	190	50	40	0.6
PLA20HA	180	60	80	0.6
PO	290	110	30	0.8
PO30TiO	290	110	30	0.8
PO40TiO	290	110	30	0.8

**Table 3:** Mean T_g_ and T_m_ (±SD) values of each filament after the second heating scan.

Material	T_g_ (°C)	T_m_ (°C)
ABS	111.4 ± 3.1	/
PLA	58.8 ± 3.2	150.8 ± 1.0
PLA50ZrO_2_	58.5 ± 0.7	/
PLA20HA	46.6 ± 2.7	151.6 ± 2.7
PO	135.0 ± 1.0	/
PO30TiO	134.0 ± 1.9	/
PO40TiO	147.1 ± 14.7	/

**Table 4:** Mean value ± standard deviation of the mechanical properties of the studied materials.

	Tensile Strength (MPa)	Maximum Deformation (%)	Young’s Modulus (MPa)
ABS	37.3 ± 2.3	9.1	1602 ± 55
PLA	48.4 ± 4.0	9.9	2266 ± 146
PLA50ZrO_2_	4.2 ± 1.5	2.7	369 ± 17
PLA20HA	29.4 ± 0.5	1.3	2489 ± 46
PO	40.4 ± 1.6	2.8	1294 ± 126
PO30TiO	18.9 ± 0.8	4.7	926 ± 21
PO40TiO	15.8 ± 0.3	2.8	953 ± 48

**Table 5:** Density measurements and porosity (%) before and after printing.

Materials	Density (g/cm^3^)		Porosity (%)
	FILAMENTS	BRICKS	FILAMENTS–BRICKS
ABS	1.047	1.0335	1.29
PLA	1.281	1.2476	2.61
PLA50ZrO_2_	3.506	3.3455	4.58
PLA20HA	1.537	1.3716	10.77
PO	0.992	0.9670	2.52
PO30TiO	1.478	1.4770	0.07
PO30TiO	1.918	1.8550	3.28

**Table 6:** Relative permittivity (ε_r_) and dielectric loss tangent (tan δ) values of printed samples.

Bricks	ε_r_	τan δ
ABS	2.58	0.0055
PLA	3.00	0.0119
PLA50ZrO_2_	8.16	0.0073
PLA20HA	3.66	0.0205
PO30TiO	3.64	0.0006
PO40TiO	5.86	0.0007

**Table 7:** Parameters used on CST studio for all the experimental materials, as well the resonant frequency and minimum of |S_11_| results after simulation of the dielectric resonator prototypes.

Materials/ Parameters	ABS	PLA	PLA50ZrO_2_	PLA20HA	PO30TiO	PO40TiO
r_DR_	32 mm	24 mm	20 mm	22 mm	22 mm	23 mm
h_DR_	38 mm	30 mm	22 mm	26 mm	28 mm	27 mm
l_GP_	200 mm	200 mm	200 mm	200 mm	200 mm	200 mm
h_GP_	1 mm	1 mm	1 mm	1 mm	1 mm	1 mm
d_fp_	22 mm	22 mm	18 mm	20 mm	20 mm	18 mm
h_f_	18 mm	16.5 mm	15 mm	15 mm	15 mm	16 mm
r_c_	2 mm	2 mm	1.5 mm	2 mm	2 mm	2 mm
ε_r_	2.58	3.01	8.16	3.66	3.64	5.86
**Results**						
Frequency	2.46 GHz	2.47 GHz	2.44 GHz	2.48 GHz	2.45 GHz	2.42 GHz
Minimum of |S_11_|	−36 dB	−26 dB	−23 dB	−35 dB	−31 dB	−25 dB

**Table 8:** Resonant frequency, the minimum of the reflection coefficient (|S_11_|), and −10 dB BW results were obtained after VNA measurements for all the prototypes.

Materials	VNA Experimental Measurements		
	Frequency (GHz)	Minimum of |S_11_| (dB)	BW (−10 dB) (%)
ABS	3.27	−16	34
PLA	3.23	−21	31
PLA50ZrO_2_	2.22	−25	17
PLA20HA	3.43	−20	38
PO30TiO	2.61	−12	36
PO40TiO	2.02	−18	16

**Table 9:** Fundamental properties comparison among the materials for the selection of prototype in sensing application.

Materials	Dimensions of Prototypes (Volume, mm^3^)	Dimensions of Prototypes (Volume, λ_0_^3^)	Printability	Cost (EUR/Prototype)	BW (−10 dB) (%)
ABS	122 × 10^3^	160 × 10^−3^	Easy	2.22	34
PLA	54 × 10^3^	67 × 10^−3^	Easy	1.39	31
PLA50ZrO_2_	28 × 10^3^	11 × 10^−3^	Medium	58.75	17
PLA20HA	40 × 10^3^	60 × 10^−3^	Medium	212.86	38
PO30TiO	43 × 10^3^	28 × 10^−3^	Medium	19.08	36
PO40TiO	45 × 10^3^	14 × 10^−3^	Medium	30.17	16

## Data Availability

The original contributions presented in the study are included in the article, further inquiries can be directed to the corresponding author.
